# In Vitro Modulation of Macrophage Inflammatory and Pro-Repair Properties Essential for Wound Healing by Calcium and Calcium-Alginate Dressings

**DOI:** 10.3390/cells14120909

**Published:** 2025-06-16

**Authors:** Yara Adib, Kevin Serror, Jose Amaya Pinzon, Laura Duciel, Marine Delagrange, Bertrand Ducos, David Boccara, Maurice Mimoun, Marc Chaouat, Armand Bensussan, Marina Samardzic, Martine Bagot, Céline Des Courtils, Laurence Michel

**Affiliations:** 1 Inserm UMRS_1342, Hôpital Saint-Louis, 1 Avenue Claude Vellefaux, 75010 Paris, France; yaraadib7@gmail.com (Y.A.); kevin.serror@aphp.fr (K.S.); jaamayap@unal.edu.co (J.A.P.); armand.bensussan@inserm.fr (A.B.); 2 UFR de Sciences du Vivant, Université Paris Cité, 75006 Paris, France; martine.bagot@aphp.fr; 3 Laboratoires Brothier, 92735 Nanterre, France; laura.duciel@brothier.com (L.D.); marina.samardzic@brothier.com (M.S.); celine.des-courtils@brothier.com (C.D.C.); 4 Service de Chirurgie Plastique, Reconstructive et Esthétique, Hôpital Saint-Louis, 75010 Paris, France; dacboc9@hotmail.com (D.B.); maurice.mimoun@aphp.fr (M.M.); marc.chaouat@aphp.fr (M.C.); 5 High Throughput qPCR Core Facility, École Normale Supérieure, Université Paris Sciences & Lettres, 75005 Paris, France; marine.delagrange@bio.ens.psl.eu (M.D.); bertand.ducos@bio.ens.psl.eu (B.D.); 6 Service de Dermatologie, APHP, Hôpital Saint Louis, 75010 Paris, France

**Keywords:** macrophage, polarization, calcium alginate, wound healing

## Abstract

Macrophages participate in cutaneous wound healing by adopting M1 pro-inflammatory and M2 immunoregulatory/pro-repair phenotypes. Chronic wounds associated with a deficient macrophage response could benefit from treatments that restore an acute inflammatory response and promote healing. Calcium-alginate dressings release calcium ions, which are potent bioactivators of macrophage function in wounds. Here, the effects of two calcium-alginate dressings, Algosteril^®^ (ALG, pure Ca^2+^ alginate) and Biatain^®^ Alginate (BIA, Ca^2+^ alginate with carboxymethyl cellulose), and a 3 mM CaCl_2_ solution were compared in human macrophages polarized to M1 or M2. ALG and CaCl_2_ preserved monocyte viability, and BIA reduced it. Both alginates and CaCl_2_ reinforced the M1 pro-inflammatory transcriptional profile and phenotype, with significant increases in IL-6 and TNF-α secretion by ALG only. In M2 macrophages, all conditions increased the M1-specific gene expression and reduced M2 markers, suggesting an orientation toward an inflammatory profile. Only ALG significantly increased the secretion of CCL18 and VEGF, suggesting pro-repair activity. All conditions increased M2 phagocytic activity. This work demonstrates the interest in calcium alginates for stimulating macrophage subtypes, which could help restore wound healing, especially in patients with compromised innate immunity. It highlights the differences among the calcium-alginate dressings. The pure alginate shows higher stimulation of macrophage pro-inflammatory and pro-repair functions.

## 1. Introduction

Macrophages, including tissue-resident and blood-derived monocytes, are innate immune cells that mature under the influence of signals from the local micro-environment. The role of macrophages in modulating immune responses has garnered significant attention due to their remarkable capacity to alter phenotypes in response to various stimuli [1]. Such macrophage plasticity enables these immune cells to adapt to their microenvironments and exhibit diverse responses to specific challenges. According to the environmental stimuli, non-polarized mature macrophages (M0) polarize into more inflammatory (M1) or immunoregulatory (M2) macrophages, two activation states with distinctive cellular morphologies, surface receptor expression, gene signatures, cytokine profiles, phagocytosis efficiency, and capacity for antigen presentation to T-cells [2]. In addition to the traditional M1 and M2 phenotypes, different populations of macrophages have been recently recognized with novel phenotypes, including the M3, M4, M17, Mmox, Mhem, M(Hb), and HA-Mac phenotypes, exhibiting several specific properties [1]. The adaptive potential of these subtypes positions them as active regulators of immune responses and disease progression. According to their phenotypes, they can be involved in tumors, atherosclerotic lesions, allergic inflammation, and autoimmune diseases but are not relevant for wound healing [1].

M1 and M2 macrophages are key players in cutaneous wound healing throughout all its phases. The M1 pro-inflammatory macrophages predominate during the early phase of wound healing, the inflammatory phase. They express high levels of co-stimulatory molecules (CD80 and CD86) and major histocompatibility complex (MHC) class II, produce pro-inflammatory cytokines (TNF-α, IL-1β, IL-6, IL-8, etc.), and display strong microbicidal activities by producing reactive oxygen species (ROS) and nitric oxide (NO). M1 macrophages are crucial to promote the acute immune response necessary to fight infection and clean the wound bed. In the subsequent proliferation and tissue remodeling phases, macrophages transition toward an anti-inflammatory and pro-repair M2 phenotype [2]. M2 macrophages exhibit a high phagocytic capacity for microbes, apoptotic cells, and cell debris [3]. They present an anti-inflammatory profile with the expression of CD200R, CD163, or CD206, in particular, and produce anti-inflammatory and pro-repair cytokines, such as IL-10, TGF-β, or VEGF and immuno-regulatory factors including CCL18 and CCL22. M2 macrophages participate in inflammation resolution and promote granulation tissue formation and wound closure.

Chronic wounds, including venous leg ulcers (VLUs), diabetic foot ulcers (DFUs), and pressure ulcers, represent a major healthcare burden in modern societies. They are associated with aging, vascular diseases, diabetes, and others. Chronic wound treatments are still inefficient with 50% DFUs and 70% VLUs failing to heal [4]. Prolonged, exacerbated inflammation has long been considered the cause of a wound becoming chronic. This paradigm has shifted, as recent studies associate wound healing defects with deficient innate immunity response, notably in diabetic and elderly patients. In particular, defects in macrophage function have been reported in chronic wounds in the elderly and diabetic foot ulcers [4,5,6,7,8]. In non-healing vs. healing DFUs, decreased macrophage recruitment, decreased macrophage phagocytic capability, and an increased proportion of M2-like macrophages have been reported, stressing the role of ineffective, low-grade inflammation in slowing or stalling wound healing [4,9]. These studies highlight the need to boost and restore an acute inflammatory response to promote healing of chronic wounds.

For complex wound treatment, dressing selection is essential, as inappropriate dressings can delay wound healing [10]. Modern wound dressings favor wound healing through their physical properties, which provide a moist environment and manage excess exudate. The upcoming innovation challenges in wound dressings include incorporating active ingredients or cells, such as macrophages, to specifically act on the defective cellular microenvironment of chronic wounds [11,12]. Until now, few such bioactive dressings have been developed and are available for patients. This is the case with alginate dressings, which release calcium ions, a potent cell activator [13].

These dressings are made of alginate, a natural polysaccharide extracted from brown algae composed of guluronic (G) and mannuronic acid (M) units, bound with calcium ions. Alginate dressings vary in their composition; some are made of 100% calcium alginate, some are mixed with carboxymethyl cellulose (CMC) or sodium alginate, for instance [14,15]. The M/G ratio of their alginate fraction also varies considerably, depending on numerous parameters (the species of the algae selected, their geographic origin, the harvesting season, etc.) [16]. The M/G ratio influences Ca^2+^ chelation by alginate and its release into the wound [17]. Thus, the performance and biological activity of various alginate dressings differ significantly, depending on their composition, the chemistry of their alginate fraction, and their calcium release [18,19,20].

The effect of an alginate polymer on the regulation of macrophage functions is unclear, as both pro-inflammatory and anti-inflammatory effects have been reported [17,18,21,22,23]. These effects vary notably depending on the M/G ratio of the alginate [21,22,24,25]. It has also been demonstrated that calcium signaling stimulates macrophage pro-inflammatory cytokine and ROS production, as well as phagocytosis [26,27,28,29]. Calcium-alginate dressings thus appear as a relevant treatment strategy to boost macrophage function and restore an efficient inflammatory phase, especially in chronic wounds. In this regard, a previous study on a mixed Ca^2+^ and Na^+^ alginate dressing reported stimulation of macrophage pro-inflammatory functions (TNFα and IL-1β secretion) [30]. However, the performance of one alginate dressing cannot be transposed to the others, and there is a need to evaluate and compare them [18]. To our knowledge, no study has compared the effects of commercially available alginate dressings on macrophages, despite their essential functions in wound healing. This study evaluated the modulation of M1 and M2 macrophage activities by the following two calcium-alginate dressings of different compositions: Algosteril^®^ (ALG, pure calcium alginate) and Biatain^®^ Alginate (BIA, calcium alginate + CMC). The effects of the dressings on macrophages were analyzed using dressing-conditioned media to extract components released by the dressings that diffuse to the cells in the wound, in accordance with ISO 10993-12 standard [31] for medical device evaluation. These effects were compared to those induced by a CaCl_2_ solution to assess the specific contribution of Ca^2+^ to the overall impact of the dressings. Macrophage morphology, gene and cytokine profiles, surface expression markers, and their phagocytic activity were analyzed. Paracrine factors secreted by macrophages were further studied for their effects on dermal fibroblasts in the presence or absence of alginate dressings or CaCl_2_ solution.

## 2. Materials and Methods

### 2.1. Preparation of Conditioned Medium from Calcium-Alginate Dressings and a CaCl_2_ Solution

Conditioned media (CM) from a pure calcium-alginate dressing (ALG, Algosteril^®^, Laboratoires Brothier, 41 Rue de Neuilly, Nanterre, France) and a calcium-alginate dressing composed of 85% Ca^2+^ alginate and 15% carboxymethyl cellulose (CMC) (BIA, Biatain^®^ Alginate, Coloplast, Humlebaek, Denmark) were prepared according to the European recommendations of ISO 10993-12, as follows: 1 cm^2^ of dressing was incubated at 37 °C for 24 h in 1 mL of culture medium (Gibco, Billings, MT, USA) composed of Roswell Park Memorial Institute medium RPMI 1640, supplemented with 10% fetal bovine serum (FBS), 1% L-glutamine, and 1% penicillin–streptomycin (complete R10 medium). To remove residues of the wound dressing fibers, conditioned media were filtered twice using 0.45 µm filters (VWR International, Radnor, PA, USA). The control CM (Cont) was prepared by a 24 h lasting incubation of complete R10 medium at 37 °C. CM were stored at 4 °C for a maximum of one week and used half-diluted in all experiments. A sterile Ca^2+^-containing stock solution was prepared at 2.4 M by adding calcium chloride, CaCl_2_.2H_2_O (Sigma, Winston Park Drive Oakville, ON, Canada), into deionized water diluted in complete R10 medium to a final concentration of [3 mM] Ca^2+^. The 3 mM concentration was chosen to be in the range of the concentration of Ca^2+^ released by the studied alginate dressing, as previously described [19]. At this concentration, the chloride ion supply is negligible compared to the physiological chloride ion concentration (approximately 150 mM). Herein, the concentrations of calcium ions released in the control CM and in the CM of alginate dressings over a 24-hour incubation at 37 °C were also measured using inductively coupled plasma/atomic emission spectrometry, according to the NF EN ISO 11885 standard [32]. The concentrations of calcium ions released in the control CM and in the CM of the alginate dressings of the repeated samplings over the experiments, reached a mean range of 0.61 ± 0.06, 3.61 ± 0.09, and 4.1 ± 0.08 mM (mean ± SEM) for complete R10 medium, ALG-CM, and BIA-CM, respectively.

### 2.2. Human Monocyte Isolation and Purification

Human peripheral blood mononuclear cells (PBMCs) were isolated from buffy coats of healthy donors obtained from a blood bank facility (EFS Paris-France, under an academic official convention #CCPSL 2024-2027-001) by centrifugation on a Ficoll density gradient (d = 1.077 g/mL, lymphocyte separation medium, Eurobio Scientific, Avenue de Scandinavie, ZA de Courtaboeuf, Les Ulis, France). Classical monocytes CD14^+^CD16^−^ were purified using a negative immunomagnetic selection according to the manufacturer’s protocol (Classical Monocyte Isolation Kit, Miltenyi Biotec, Unit 11, 2 Eden Park Drive, Macquarie Park, NSW, Australia).

### 2.3. Monocyte Differentiation and Polarization In Vitro

**Scheme 1:**
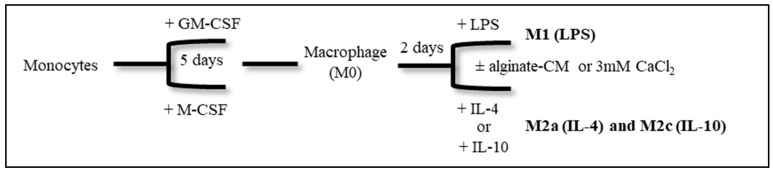
The experimental design for macrophage polarization.

After mononuclear cell purification from blood, CD14^+^CD16^−^-isolated monocytes were differentiated into mature M0 macrophages by culture for 5 days at a concentration of 0.5 × 10^6^/mL in a complete R10 medium at 37 °C in a humidified incubator under a 5% CO_2_ atmosphere supplemented with 50 ng/mL of either granulocyte-macrophage colony-stimulating factor (rhGM-CSF, Peprotech, Rocky Hill, NJ, USA) or macrophage colony-stimulating factor (rhM-CSF, Peprotech, Rocky Hill, NJ, USA) depending on their further polarization into M1 or M2 subtypes, respectively. On day 5, macrophage M0 adherent cells were washed and polarized for an additional 48 h in the presence of either (1) 100 ng/mL of lipopolysaccharide (LPS) purified from *Escherichia coli* O55:B5 (Enzo, Fisher Scientific SAS, Illkirch Graffenstaden, France) (M1 phenotype), (2) 20 ng/mL of rhIL-4 (Peprotech) (M2a phenotype), (3) 20 ng/mL of rhIL-10 (Peprotech, Rocky Hill, NJ, USA) (M2c phenotype), or (4) left unstimulated for the duration of the 2 days of culture (M0 macrophages), as detailed in the experimental Scheme 1. During these two days of culture, Cont, ALG, BIA, and CaCl_2_ were also added to assess the effect of the alginate-conditioned medium or calcium solution on macrophage polarization. On day 7, bright field images were taken for morphological feature evaluation using an EVOS XL Core microscope with a magnification of 20×. Supernatants were collected and stored at −80 °C for further cytokine measures. Adherent cells were detached for phenotypic characterization by flow cytometry analysis and gene expression detection after cell lysis before RNA extraction.

### 2.4. MTT Assay

A total of 4 × 10^4^ human monocytes were seeded in a 96-well plate in complete R10 medium. Twenty-four hours after seeding, the Cont, ALG, BIA, and CaCl_2_ were individually added to wells. Each sample was run in triplicate. The plates were maintained at 37 °C in 5% CO_2_ for 5 days. Four hours before the end of the incubation time, 20 µL of 3-(4,5-dimethylthiazol-2-yl) 2,5diphenyltetrazolium bromide solution (MTT, Sigma-Aldrich, St. Louis, MO, USA, 5 mg/mL) was added to each well. After 4 h of incubation, plates were centrifuged, and the medium was removed from each well. Precipitated formazan crystals were dissolved by adding 200 µL of dimethyl sulfoxide (DMSO, Sigma-Aldrich, Saint Louis, MO, USA) to each well. The microplate was shaken at room temperature for 10 min and prepared for reading using a microplate reader at 550 nm.

### 2.5. RNA Isolation and cDNA Synthesis

The RNA was extracted using the RNeasy Kit (Qiagen, Hilden, Germany) according to the manufacturer’s protocol. The RNA concentration and purity were determined by UV spectrophotometry (NanoDrop Technologies, Wilmington, DE, USA). Total RNA was reverse transcribed to cDNA using the Reverse Transcription Master Mix (Fluidigm, San Francisco, CA, USA) according to the manufacturer’s instructions.

### 2.6. Gene Expression

#### 2.6.1. Real-Time Quantitative PCR (RTq-PCR)

As M1 or M2a/M2c macrophages express specific non-shared genes, the expression of these genes was analyzed by RT-qPCR using SYBR Select Master Mix (Thermo Fisher Scientific, Waltham, MA, USA) for 50 cycles on a Lightcycler 480 (Roche, Basel, Switzerland). Relative mRNA expression was calculated based on the 2^−Δ^^C^^t^ method, using the β2M housekeeping gene as reference. Relative mRNA expression ratios were calculated by taking the respective Cont of each phenotype as 1. Sequences of transcript primers are listed in Table 1.

#### 2.6.2. Microfluidic qPCR

Specific target amplification (STA) was first performed to increase the number of target molecules in the samples and standards (NTCs, negative RT controls, and RT calibration samples). Microfluidic qPCR was then conducted using the BioMark HD Real-Time PCR system in a 48.48 DynamicArray integrated fluidic circuit (IFC; Fluidigm Corporation, San Francisco, CA, USA). The IFC was loaded according to the manufacturer’s instructions with the assay mix consisting of assay loading reagent and primer mix (forward and reverse) and a sample pre-mix combining EvaGreen^®^ Supermix with low ROX (Biorad, Cressier, Switzerland) and a volume of each sample. The primer pairs are listed in Table 1. The loaded IFC was transferred to the BioMark instrument before qPCR was conducted using the following conditions: 50 °C for 2 min, 95 °C for 10 min, and then 40 cycles of 95 °C for 15 s, 70 °C for 5 s, and 60 °C for 1 min. Data were assessed using the Fluidigm Real-Time PCR software (PN 100-2637). The threshold fluorescence intensity was manually set for each assay based on the logarithmic view of the amplification curve to obtain quantification cycle (Cq) values. The Real-Time PCR Analysis Software (PN 100-2637). flags all reactions that do not conform to the selected thresholds (i.e., low quality score, multiple or no melting curve peaks, or reactions where the normalized fluorescence is below the threshold).

Samples were considered quantifiable if the measured quantity was greater than the observable quantity in the lowest concentration standard (i.e., limit of quantification; LOQ) and the background signals in the NTC (if any). Samples were considered non-detected (nd) if no amplification was observed within 40 cycles. Relative mRNA expression was calculated based on the 2^−(Cq gene of interest − Cq housekeeping gene)^ method using β-actin as the best housekeeping gene tested among two others. Relative mRNA expression ratios were calculated by taking the respective Cont of each phenotype as 1. Differentially expressed genes (DEGs) with fold changes of an increase or decrease greater/less than or equal to 1.5 were considered for further analysis. A comparative heatmap was assessed according to the 34 analyzed genes with an extended cluster analysis, as well as without genes that were detected in only one subset of macrophages, such as GM-CSF, IL-1β, IL-8, IL-6 in M1, TGF-β, and FABP4 in M2a and IL-10 in M2c.

### 2.7. Phenotypic Features Analysis by Flow Cytometry

Polarized macrophages on day 7 were harvested by 0.05% Trypsin-EDTA (Gibco), centrifuged, counted, and incubated with Fc-blocking solution (Invitrogen, Carlsbad, CA, USA). Cells were stained with the following cocktail of fluorochrome-conjugated mAbs (Invitrogen): CD14-Super Bright 600, CD80-PE, CD86-Super Bright 436, TLR4-APC, CD200R-PerCP-eFluor 710, CD163-Super Bright 645, CD36-FITC, and Fixable Viability Dye eFluor 506 in staining phosphate-buffered saline (PBS) supplemented with 0.5% of bovine serum albumin (BSA) and 2 mM of ethylene diamine tetraacetic acid (EDTA) (Invitrogen). Data were acquired on an LSR Fortessa flow cytometer (BD Biosciences, San Jose, CA, USA) and analyzed using FlowJo software version 10 (BD Biosciences, San Jose, CA, USA). After gating out dead cells, macrophages were identified by CD14 expression, and surface marker expression levels were studied among positive CD14 cells.

### 2.8. Cytokine Production by ELISA

The detection of TNF-α (Peprotech, Rocky Hill, NJ, USA), IL-6 (Peprotech, Rocky Hill, NJ, USA), CCL18 (Biotechne, Minneapolis, MN, USA), and VEGF (Peprotech, Rocky Hill, NJ, USA) protein levels in cell culture supernatants was measured using sandwich ELISA kits according to the manufacturer’s instructions. Briefly, 96-well plates were pre-coated with capture antibodies, followed by incubation with cell culture supernatants, biotinylated detection antibodies, and streptavidin–HRP. Color development was achieved using an ABTS substrate (TNF-α, IL-6, VEGF) or TMB substrate (CCL18), and the absorbance was measured at 405 nm (TNF-α, IL-6, VEGF) or 450 nm (CCL18) using a microplate reader. Cytokine concentrations were calculated using standard curves generated with recombinant proteins. All samples were assayed in duplicate, and the results are reported in pg/mL.

### 2.9. IncuCyte^®^ Phagocytosis Assay

To examine the phagocytic activity, polarized macrophages (1 × 10^4^ cells/per triplicate) were plated in a 96-well, flat, and clear-bottomed plate and allowed to adhere for 2 h. Cells were then co-incubated with 2.5 µg/mL of pHrodo^TM^ Green *E. coli* Bioparticles Conjugate (Thermo Fisher Scientific), reconstituted in complete R10 medium according to the manufacturer’s instructions. The plates were transferred into a cell incubator equipped with the IncuCyte^®^ platform. A whole well image was taken every 2 h for 3 days, and then the green object integrated density was analyzed using the IncuCyte^®^ Basic software version 2022B. The settings were fixed based on those described by Kapellos et al. [33].

### 2.10. Human Dermal Fibroblast (HDF) Cell Culture and Stimulation with M0, M1, and M2 Macrophage Supernatants

Fibroblasts migrated out of skin explants from breast reduction plastic surgery were cultured in complete R10 medium at 37 °C in a humidified incubator under a 5% CO_2_ atmosphere. Cells were amplified, used between the first and third passages, and seeded at 0.3 × 10^6^/cm^2^ density in complete R10 medium. The next day, the culture medium was replaced with supernatants from M0, M1, and M2 macrophages. After 24 h of stimulation, cells were lysed using Qiagen RLT plus buffer from the Qiagen RNeasy kit and conserved for fibroblast-associated gene analysis by RT-qPCR.

### 2.11. Statistical Analysis

The results are expressed as the mean ± SEM from independent experiments. Wilcoxon paired test were performed using Prism (GraphPad 9.1.1), as follows: § < 0.1 and * *p* < 0.05 and ** *p* < 0.01. The heatmap was generated using RStudio 2023.06.0+421, “Mountain Hydrangea” Release, for Windows.

## 3. Results

### 3.1. Monocyte Viability and Macrophage Morphology in the Presence of Alginate Dressings and CaCl_2_

**Figure 1:**
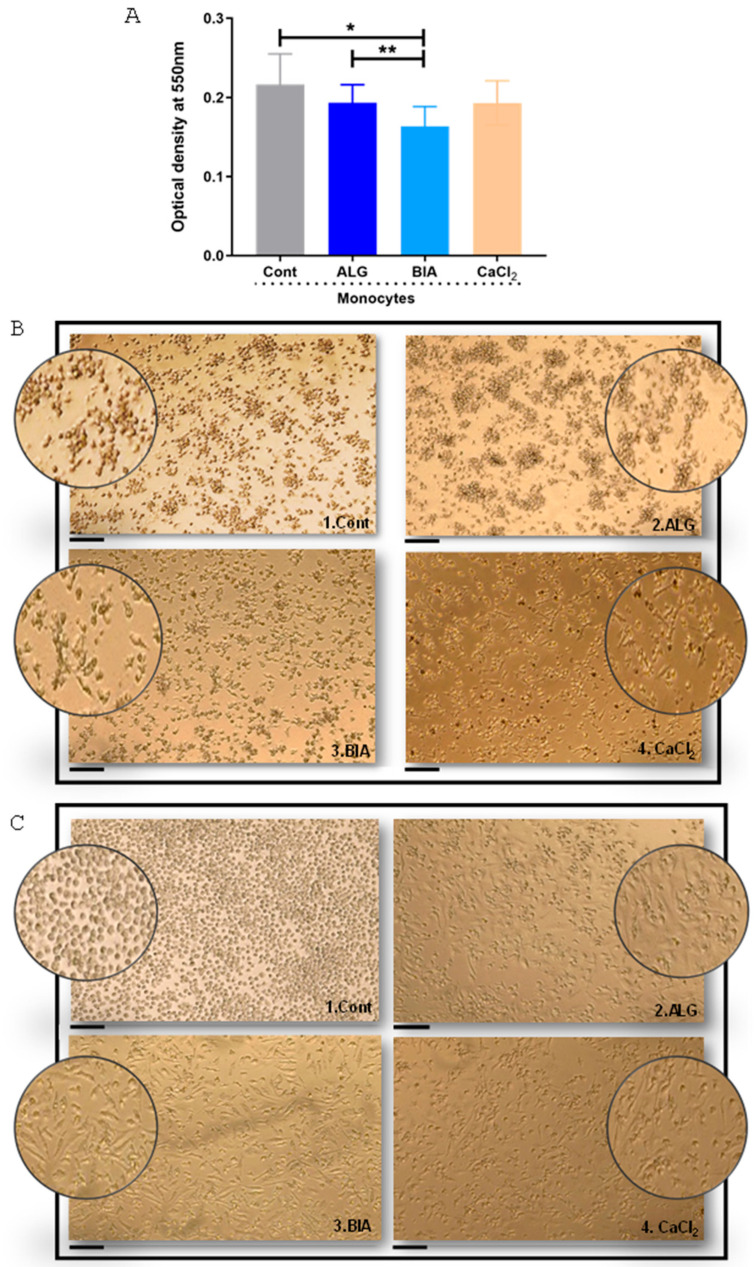
Effects of calcium-alginate-conditioned media and 3 mM CaCl_2_ solution on monocyte viability and macrophage morphology. (**A**) Monocyte viability was assessed by MTT assay after incubation for 5 days with Cont (grey bar), ALG (dark blue bar), BIA (light blue bar), and CaCl_2_ (nude bar). Bar graphs are the mean of 8 independent experiments ± SEM, with three replicates of each condition per experiment. Statistical analysis was performed using the Wilcoxon paired test; * *p* < 0.05 and ** *p* < 0.01. (**B**,**C**) Monocytes were differentiated in 5 days into mature M0 macrophages with 50 ng/mL of GM-CSF for the M1 phenotype or 50 ng/mL of M-CSF for the M2 phenotype, then polarized for 2 days to (**B**) M1 macrophages in the presence of 100 ng/mL of LPS or (**C**) M2a macrophages in the presence of 20 ng/mL of IL-4, with the control (images B1 and C1), ALG (images B2 and C2), BIA (images B3 and C3), or CaCl_2_ (images B4 and C4). Bright field images were taken after 7 days of culture. An insert showing a 2-fold size increase is provided for each condition (1B and 1C). Scale bars, 100 μm.

Monocyte viability was not affected by the presence of ALG-conditioned medium and CaCl_2_ solution, whereas it significantly decreased with the BIA-conditioned medium compared with the control (Cont) and ALG (Figure 1A).

Slight visual differences were observed in the cell shape and distribution between the M1 and M2 subtypes. The M1 macrophages mainly showed a rounded morphology, except for some rare spindle-elongated cells (Figure 1B), while the M2 macrophages presented a fully rounded cell morphology, regardless of their sub-phenotype, M2a or M2c (only M2a is presented in Figure 1C). When alginate dressings and CaCl_2_ were added during the LPS-induced polarization of the M1 macrophages, the cell morphology did not change with ALG (Figure 1B image 2), whereas it was altered with BIA or CaCl_2_ in comparison with the Cont, showing a mixed rounded and spindle-elongated morphology, with 10% and 15% of the spindle-elongated shape with the BIA and CaCl_2_, respectively (Figure 1B images 3 and 4). The addition of alginate dressings or CaCl_2_ during M2 polarization also modified the cell morphology, showing a more elongated, stretched morphology than the M2 control, with 60, 80, and 70% of the spindle-elongated shape appearing with ALG, BIA and CaCl_2_, respectively (Figure 1C images 2, 3 and 4).

### 3.2. Clustering Analysis of M1, M2a, and M2c Macrophages Polarized in the Presence of Alginate Dressings or CaCl_2_

**Figure 2:**
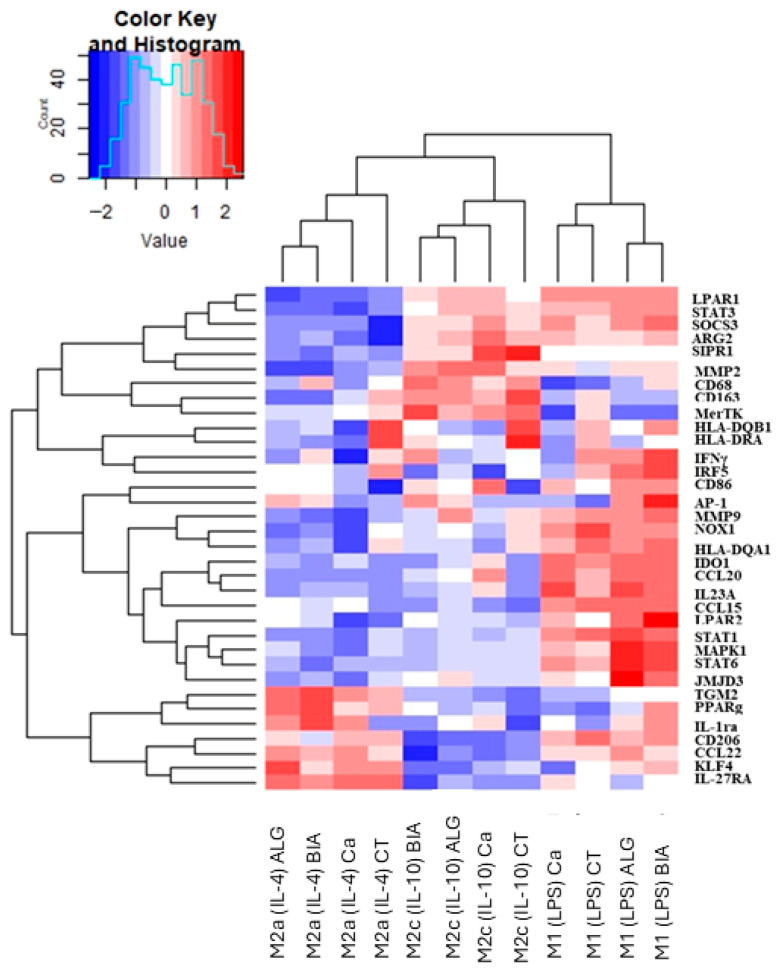
Heatmap/transcriptional profiles of the M1, M2a, and M2c macrophages polarized in the presence of alginate-CM and CaCl_2_ solution at 3 mM. Heatmap of the mean expression and hierarchical clustering of 34 macrophage-related genes in M1 (n = 8), M2a (n = 7), and M2c (n = 6).

A comparative transcriptional profile heatmap was generated with an extended cluster analysis of 34 macrophage lineage-related genes (Figure 2). The gene clustering validated the phenotype of each polarized macrophage subset at the transcriptomic level. Exposure of M1 and M2 macrophages to the alginate dressings or CaCl_2_ globally preserved their respective transcriptional profiles, as observed by the conserved homogenous clustering of each subtype, although the M1 phenotype appeared strengthened upon exposure to alginate dressings.

It is noteworthy that some genes were not inserted into the heatmap representation because they were detected in only one subset of macrophages, such as GM-CSF, IL-1β, IL-8, IL-6 in M1; TGF-β and FABP4 in M2a; and IL-10 in M2c.

**Figure 3:**
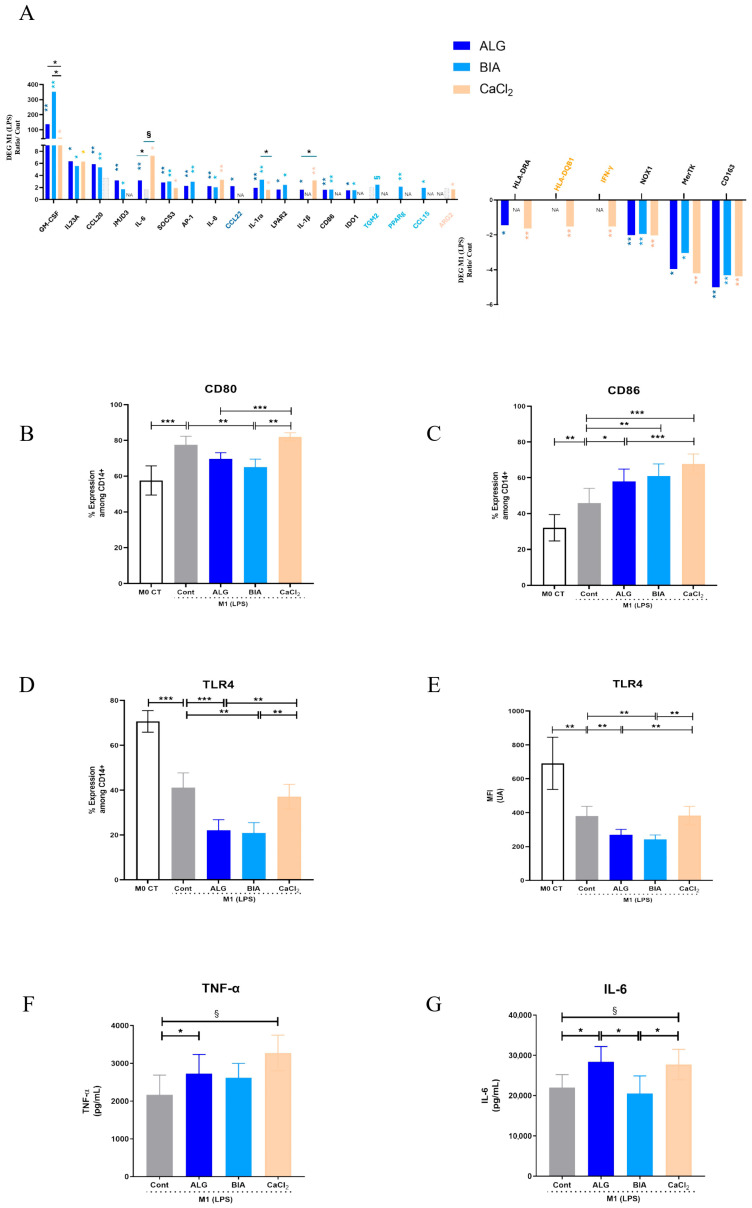
M1 macrophage characteristics. (**A**) Differentially expressed genes (DEGs) in M1 under ALG (dark blue bars), BIA (light blue bar), and CaCl_2_ solution (nude bars) are presented as the fold change of the increase (≥1.5 fold) or decrease (≤1.5 fold) in mean gene expression compared with the respective phenotype Cont. Grey bars are data with a fold change ≥ 1.5 fold with no significance. Missing bars (NA) are data that do not meet the criteria. (**B**–**E**) Surface marker expression analyzed by flow cytometry of M1 macrophages polarized in the presence of the control medium (Cont grey bars), ALG (dark blue bars), BIA (light blue bars), and CaCl_2_ solution (nude bars) or non-polarized M0 (white bars): (**B**) CD80 (n = 12), (**C**) CD86 (n = 12), (**D**) TLR4 (n = 12), and (**E**) mean fluorescence intensity of TLR4. (**F**,**G**) Quantification by ELISA of (**F**) TNF-α (n = 6) and (**G**) IL-6 (n = 9) secretion levels in the M1 supernatants of macrophages polarized in the presence of Cont medium (grey bars), ALG (dark blue bars), BIA (light blue bars), and CaCl_2_ solution (nude bars). Bar graphs represent the mean ± SEM. Statistical analysis was conducted using the Wilcoxon paired test; * *p* < 0.05, ** *p* < 0.01, and *** *p* < 0.001; § tendency *p* < 0.1.

**Figure 4:**
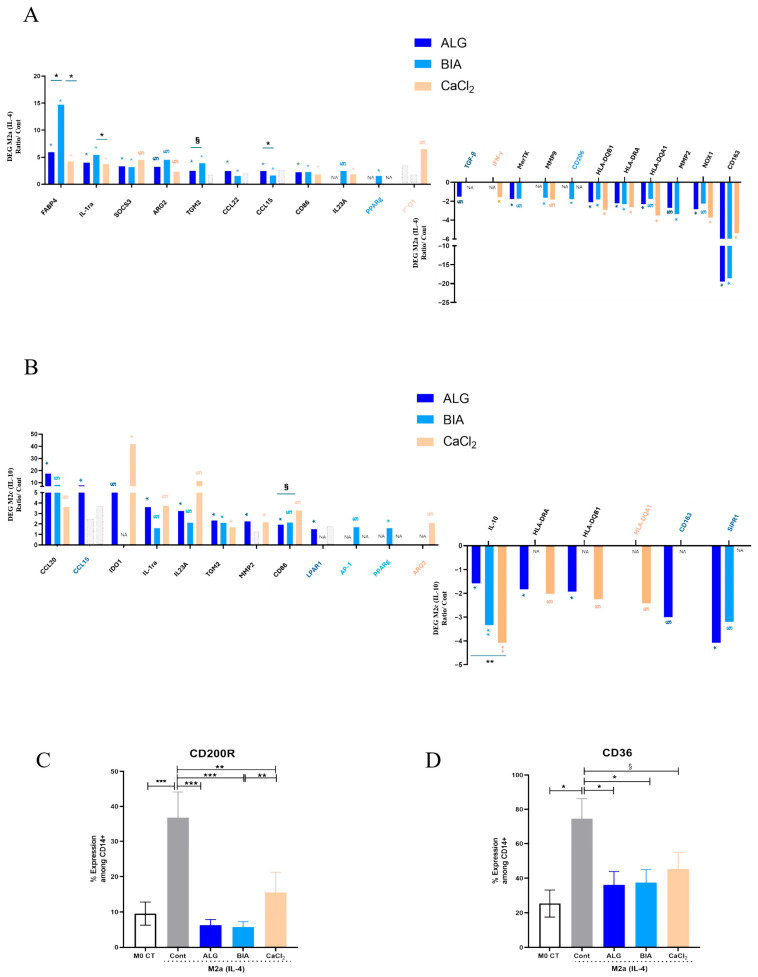

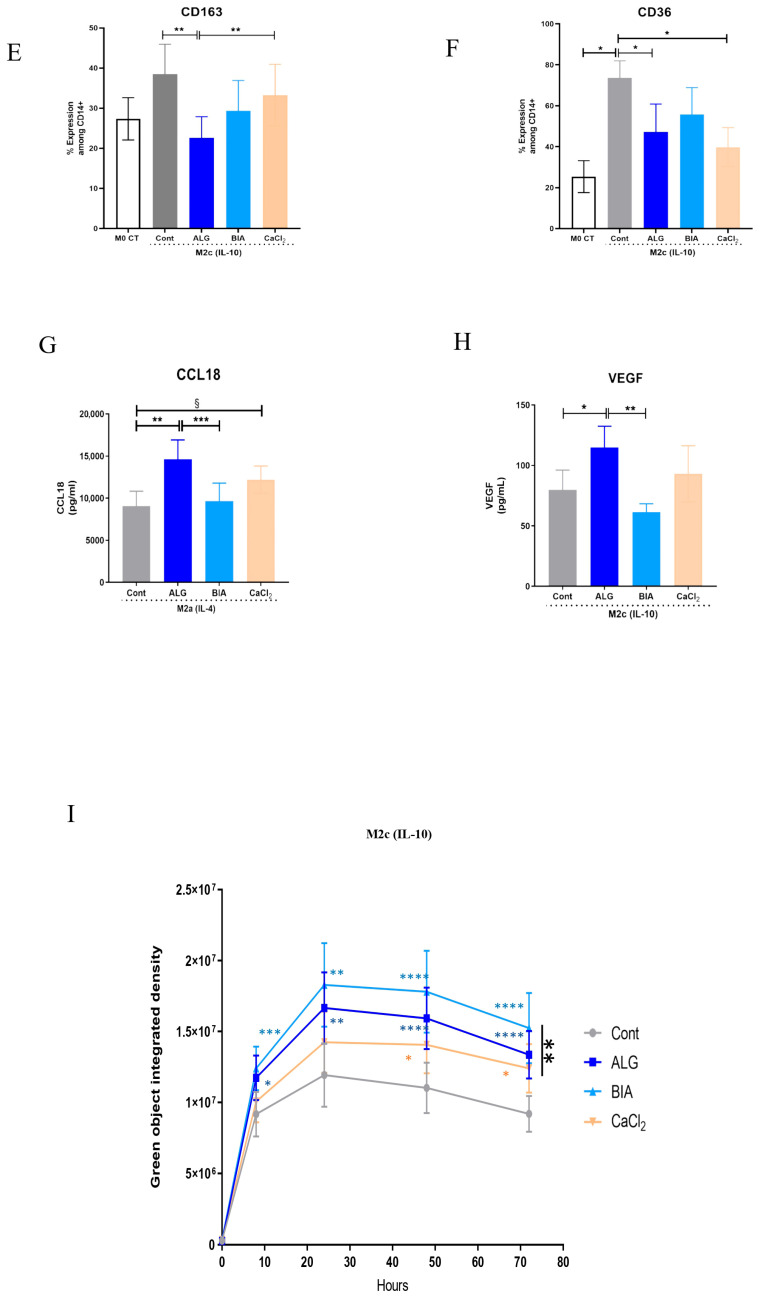
M2 macrophage characteristics. (**A**,**B**) Differentially expressed genes (DEGs) in (**A**) M2a and (**B**) M2c under ALG (dark blue bars), BIA (light blue bar), and CaCl_2_ solution (nude bars) are presented as the fold change of the increase (≥1.5-fold) or decrease (≤1.5-fold) in the mean gene expression compared with the respective phenotype controls (Cont). Grey bars are data with a fold change ≥ 1.5 fold with no significance. Missing bars (NA) are data that do not meet the criteria. (**C**–**F**) Surface markers expression was analyzed by flow cytometry of M2a for (**C**) CD200R (n = 12) and (**D**) CD36 (n = 7) and of M2c for (**E**) CD163 (n = 12) and (**F**) CD36 (n = 7), polarized in the presence of Cont (grey bars), ALG (dark blue bars), BIA (light blue bars), and CaCl_2_ solution (nude bars) or non-polarized M0 (white bars). (**G**,**H**) Quantification by ELISA of (**G**) CCL18 in the M2a and (**H**) VEGF in the M2c supernatants of macrophages polarized in the presence of Cont (grey bars), ALG (dark blue bars), BIA (light blue bars), and CaCl_2_ solution (nude bars). Bar graphs represent the mean ± SEM. Statistical analysis was conducted using the Wilcoxon paired test; * *p* < 0.05, ** *p* < 0.01, *** *p* < 0.001, and **** *p* < 0.0001; § tendency toward *p* < 0.1. (**I**) 1 × 10^4^ of the M2c macrophages was co-incubated with 2.5 µg/mL of pHrodo^TM^ Green *E. coli* bioparticles for 3 days. The phagocytic activity of the M2c macrophages polarized in the presence of Cont (grey circle), ALG (dark blue square), BIA (light blue triangle), and CaCl_2_ solution (nude diamond). The green object’s integrated density was determined at the following 4 time points using Incucyte analysis software 2022B: 8 h, 24 h, 48 h, and 72 h. The results are the mean ± SEM (n = 16).

Using a fold change of 1.5 and a *p*-value ≤ 0.05 as the analytical criteria, the analysis of the differentially expressed genes (DEGs) for the M1, M2a, and M2c subtypes (Figure 3A and Figure 4A,B) exposed to alginate dressings or CaCl_2_ versus the controls identified a set of 19 DEGs in M1, 18 in M2a, and 15 in M2c.

### 3.3. Focus on M1 Macrophages

Transcriptional profile—A transcriptional profile analysis of the M1 macrophages identified an increased expression of eleven genes in common between ALG and BIA, among which five genes were also shared with CaCl_2_, as presented in Table 2 and Figure 3A. Moreover, CCL22 was induced with only ALG, CCL15, and PPARγ with BIA and ARG2 with CaCl_2_.

Most of these genes are specific to the M1 phenotype (underlined genes in Table 2) and include pro-inflammatory factors or signaling pathway molecules. Concerning the decrease in gene expression in the M1 macrophages, only three genes were significantly downregulated in the presence of alginate dressings and CaCl_2_ (Table 2). HLA-DRA expression significantly decreased with both ALG and CaCl_2_, while HLA-DQB1 and IFN-γ were significantly downregulated only by the CaCl_2_ solution (Figure 3A).

Phenotype—A significant decrease in CD80 expression in polarized M1 macrophages was detected in the presence of BIA compared with the control (Figure 3B), and no alteration was observed with the ALG and CaCl_2_ solution. A significant upregulation in CD86 expression was detected in the presence of both conditioned media and CaCl_2_ solution compared with the control (Figure 3C).

M1 macrophages were also analyzed for TLR4 surface expression. It has been shown that LPS, a specific ligand of the TLR4 receptor, induces TLR4 activation through its internalization, leading to a decrease in its surface expression [34]. Accordingly, our data show that the TLR4 surface expression and mean fluorescence intensity (MFI) were significantly decreased in the control macrophages polarized with LPS compared with mature non-polarized macrophages (M0), as shown in Figure 3D,E, respectively. TLR4 expression and MFI significantly decreased under LPS polarization in the presence of ALG or BIA compared with the control macrophages polarized into the M1 phenotype with LPS alone (Figure 3D,E). In contrast, the expression of TLR4 was not affected by the addition of the CaCl_2_ solution during M1 LPS polarization.

Cytokine secretion—At the protein level, the secretion of pro-inflammatory cytokines, such as TNF-α and IL-6, by M1 macrophages significantly increased in the presence of ALG compared with the control, while a mild tendency toward increasing and no effect were observed with the CaCl_2_ solution and BIA, respectively (Figure 3F,G).

Altogether, the results demonstrate that the pro-inflammatory transcriptional profile and phenotype of the M1 macrophages were reinforced by calcium-alginate dressings and, to a lesser extent, by CaCl_2_. Functionally, cytokine secretion by M1 macrophages only significantly increased with the pure calcium alginate.

### 3.4. Focus on M2 Macrophages

Transcriptional profile—The transcriptional profile analysis of the M2a macrophages identified an increased expression of eight genes in common between ALG and BIA, among which five genes were also shared with CaCl_2_, as presented in Table 3 and Figure 4A. An increase in IL23A expression was observed with BIA and CaCl_2_, and an increase in PPARγ was specific to BIA and IDO1 to CaCl_2_ (Figure 4A). Although some genes induced by calcium-alginate dressings are specific to the M2a phenotype (underlined genes in Table 3), others were shared with the M1 phenotype (e.g., CD86, SOCS3, IDO1, IL23A, and CCL15).

In addition, seven genes were downregulated in the presence of both alginate dressings, with specific decreases in TGF-β with ALG and CD206 with BIA (Table 3, Figure 4A). The presence of CaCl_2_ induced a decrease in gene expression similar to that of alginates, with the addition of a decrease in IFN-γ gene expression.

In M2c (Table 4, Figure 4B), the expression of five genes was upregulated in the presence of alginate dressings and CaCl_2_. Specific increases in IDO1 and MMP2 expression were induced by CaCl_2_ and ALG, which also increased LPAR1 and CCL15 expression. Increases in AP-1 and PPARγ expression are depicted with BIA. A significant decrease in IL-10 expression was observed with the three conditions, whereas only ALG induced significant ones in HLA-DRA, HLA-DRQB1, and SIPR1 expression (Table 4, Figure 4B).

Phenotype—The analysis of the surface expression of specific M2a markers showed a significant reduction in CD200R expression in the presence of either alginate dressings or CaCl_2_ solution compared with the control (Figure 4C). The surface expression of CD163 on the M2c subtype significantly decreased in the presence of ALG but not in the presence of BIA or CaCl_2_ (Figure 4E). Moreover, CD36 surface expression significantly decreased with both alginate dressings on M2a and with ALG and CaCl_2_ on M2c (Figure 4D–F).

Cytokine secretion—At the protein level, M2a and M2c, respectively, secreted the immune regulatory cytokine CCL18 and the pro-angiogenic factor VEGF, and ALG significantly increased these secretions, while BIA and CaCl2 did not alter them (Figure 4G,H).

Phagocytosis—The phagocytic activity of *E. coli* bioparticles by the M2c macrophages significantly increased in the presence of ALG and BIA, as well as to a lesser extent with CaCl_2_, compared with the control (Figure 4I).

Altogether, the results demonstrate that all calcium-containing conditions stimulate phagocytosis and modulate the M2 phenotypes toward a more pro-inflammatory M1-like phenotype, with functionally significant increases in the secretion of CCL18 and VEGF with the pure calcium alginate.

### 3.5. Effects of Macrophage Paracrine Factors on Human Dermal Fibroblasts

**Figure 5:**
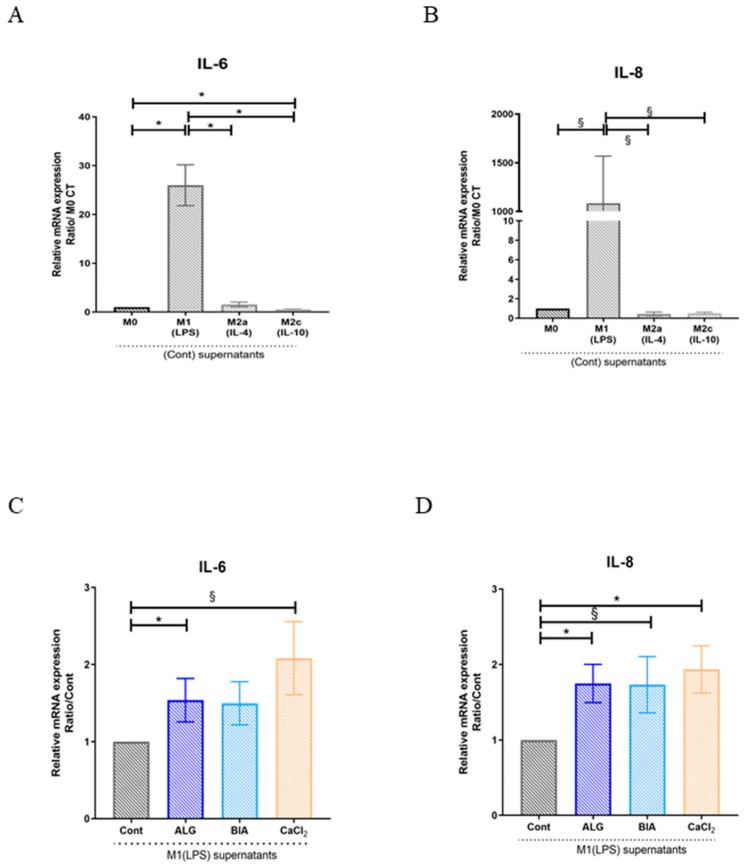
Fibroblast-associated gene expression markers. HDFs were incubated with M0 supernatants (black hatched bars), M1 (LPS) (dark grey hatched bars), M2a (IL-4) (light grey hatched bars), M2c macrophage (very-light-grey hatched bars). The relative mRNA gene expression of (**A**) IL-6 (n = 6, mean ± SEM) and (**B**) IL-8 (n = 5, mean ± SD), were studied by RT-qPCR. The relative gene expression of (**C**,**D**) IL-6 and IL-8 were analyzed for the fibroblasts incubated with supernatants from M1 polarized with LPS in the presence of ALG (dark blue hatched bars), BIA (light blue hatched bars), and CaCl_2_ (nude hatched bars). The bar graphs represent the mean ± SEM (n = 6). Statistical analysis was conducted using the Wilcoxon paired test; * *p* < 0.05; § tendency toward *p* < 0.1.

The fibroblasts stimulated for 24 h with the control M1 macrophage supernatants showed significant increases in the expression of the two major fibroblast pro-inflammatory cytokines, IL-6 and IL-8, compared with the fibroblasts stimulated with supernatants of either the M0 control or M2a and M2c macrophages (Figure 5A,B). Moreover, IL-6 and IL-8 expression also significantly increased with the supernatants from the M1 macrophages polarized in the presence of alginate dressings or CaCl_2_ compared with the fibroblasts cultured with the control M1 supernatants (Figure 5C,D). Concerning pro-repair factors, the expression of TGF-β, α-SMA, and Col1a by the fibroblasts was reduced by the M1 supernatants compared with the control M0 (Supplementary Figure S1A–C), whereas it was not affected by the supernatants from the M2 macrophages polarized in the presence, or not, of alginate dressings or CaCl_2_ (Supplementary Figure S1D–I).

## 4. Discussion

This work demonstrates that the M1 and M2 macrophages were oriented toward a more inflammatory profile by the calcium-alginate dressings, as observed at the expression and phenotypic levels. This effect is mainly recapitulated by the CaCl_2_ solution, demonstrating the role of extracellular Ca^2+^ in the effects of alginate dressings. The results highlight the differences between the two alginate dressings regarding cell viability and stimulation. Monocyte viability was preserved with ALG, whereas it was altered with BIA, and the secretion of pro-inflammatory and pro-repair factors by M1 and M2 macrophages was significantly stimulated by the pure calcium alginate.

On the M1 macrophages, the calcium-alginate dressings and Ca^2+^ reinforce the inflammatory profile at the expression and phenotypic levels. This trend is in agreement with previous studies reporting that alginate polymer and Ca^2+^ signaling potentiate the function of LPS-polarized macrophages [25,28,29,30]. In our results, this reinforcement was observed by the increase in the gene expression of several pro-inflammatory factors, including IL-1β, IL-8, IL-6, IL-23A, GM-CSF, and CCL20. In particular, GM-CSF, IL23A, and CCL20 might be responsible for activating an autocrine M1 polarization and for inducing Th17 response via the JAK-STAT pathways, and chemoattraction of various immune cell types (T-helper, T-regulatory, and immature dendritic cells) [35,36,37]. Their significant increase in the presence of calcium alginates could thus be responsible for amplifying the inflammation within the wound lesion.

The activation of the TLR4 receptor was measured by its decrease at the cell surface used as a readout of receptor internalization and endocytosis. Herein, this decrease was observed with the two alginate dressings studied but not with the CaCl_2_ solution, and this agrees with previous reports of TLR4 activation by binding alginate oligomers [25,38]. This activation of TLR4 signaling in macrophages by alginate dressings reinforces the inflammatory profile of M1 macrophages and could promote inflammation by producing IL-1β, IL-23A, and TNF-α.

Phenotypically, the M1-specific marker CD86 was overexpressed. This marker has been shown to stimulate the pro-inflammatory cytokine IL6 (a repressor of HLA-DR) and the expression of IDO (an M1 marker with immune-suppressive functions, acting through the kynurenine pathway) [39]. Consistently, in the presence of calcium-alginate dressings and Ca^2+^, HLA-DR was downregulated, and IDO was overexpressed. This could reflect a potential immunoregulatory and protective mechanism of calcium alginate in chronic inflammation [40].

Interestingly, functionally at the protein level, the secretion of the inflammatory cytokines TNF-α and IL-6 was significantly boosted by ALG but not by BIA or CaCl_2_.

Concerning M2 macrophages, alginate dressings and Ca^2+^ tended to increase the M1-specific gene expression and reduce M2-specific markers, suggesting stimulation of a more pro-inflammatory profile. The mitigation of the M2 anti-inflammatory profile was further observed via several parameters, including morphological changes, increased expression of the CCL22 and CCL15 (two cytokines that can recruit immune cells [41,42,43,44,45]), and a decrease in the M2-specific markers CD200R and CD163 at the cell surface, as well as a downregulation of the gene expression of CD163 and the anti-inflammatory cytokine IL-10 in M2a or M2c.

Nevertheless, a strengthening of factors implicated in the inflammation resolution by the M2 macrophages was induced by the alginate dressings and Ca^2+^ solution. This is exemplified by the increased gene expression of transglutaminase 2 (TGM2), FABP4, IL1ra, and Arg2 by the M2a or M2c macrophages. TGM2 and FABP4 are known to be downstream of PPARγ activation, which is considered to limit the extent of inflammation and to promote its resolution [46,47]. TGM2 is involved in efferocytosis, enabling the engulfment of apoptotic cells [48,49], and in phagocytosis, both key mechanisms in inflammation resolution by M2 macrophages. It plays a role in wound healing events, such as stabilization of the fibrin clot, recruitment of inflammatory cells to the wound area in the early stages of the inflammatory phase, and promotion of the matrix deposition, angiogenesis, and tissue remodeling [50,51]. Our results consistently demonstrate that alginate dressings and Ca^2+^ promote M2c phagocytic activity. This pro-repair effect of the alginate dressings and Ca^2+^ on the M2 macrophages is further supported by the significant decrease in CD36 at their cell surface. CD36 is a class B scavenger receptor that mediates anti-angiogenic signaling and plays a role in the promotion and severity of fibrosis [52,53,54]. Its decreased expression could, thus, have a positive effect on angiogenesis and participate in the prevention of fibrosis.

Functionally, at the protein level, only the pure calcium alginate ALG significantly increased the secretion of CCL18 and VEGF by the M2 macrophages. CCL18 is known to be implicated in the resolution of the inflammation phase, attract regulatory T cells, and induce fibroblast activation [55]. VEGF activates downstream pathways, including PI3K/AKT, MAPK/ERK, and PLCγ, and is necessary during the proliferation phase of healing as a key element in angiogenesis and granulation tissue formation. The increased secretion of this growth factor with ALG could be linked with the increased gene expression of the lysophosphatidic acid receptor LPAR1 observed in the transcriptomic analysis. Indeed, LPARs promote wound healing by acting on cell migration and proliferation, vascular development, and activation of fibroblasts into myofibroblasts, as well as VEGF secretion, IL-6, and MMP production [56,57,58].

Although the M2 macrophages showed a more pro-inflammatory (M1-like) profile in the presence of alginate dressings and CaCl_2_, they did not affect the expression of pro-repair genes (TGFβ, Col1α, and αSMA) by fibroblasts, whereas the M1 macrophages reduced it. This suggests that alginate dressings and CaCl_2_ fine tune the balance between pro-inflammatory and pro-repair macrophage activities for optimal wound healing. However, this study focused on the well-characterized M1, M2a, and M2c macrophage subtypes that play a major role in inflammation and tissue repair. Other macrophage subtypes should also be considered, and future investigations might provide a broader understanding of the immunomodulatory effects of calcium-alginate dressings.

Altogether, our results demonstrate the potential of calcium-alginate dressings to boost the inflammatory response of M1 and M2 macrophages. This suggests that the mechanisms underlying immune cell recruitment and activation, as well as microbe and debris clearance, which are necessary to resolve the inflammatory phase, could be stimulated.

The major effects were observed with the pure calcium alginate, especially at the protein level, with an increased secretion of pro-inflammatory cytokines and pro-repair factors analyzed on the M1 and M2 macrophages, respectively. These results point to the non-equivalence between the two alginate dressings studied concerning their effects on monocyte viability and macrophage stimulation, as was previously reported in other cell types [19,20]. These differences can be explained by the presence of CMC mixed with calcium alginate in BIA, with CMC having been previously shown to negatively impact some cell functions [19]. Moreover, the chemical compositions of their alginate fraction differs [18], especially their M/G ratio, which strongly influences Ca^2+^ release by alginate dressings. Extracellular Ca^2+^ is a potent cell activator via the induction of intracellular calcium signaling. This activation has been shown to require an optimal concentration of extracellular Ca^2+^ for several cell functions, as too little and too much Ca^2+^ are deleterious to their activation [13,59,60]. Previous studies have shown that the amount of Ca^2+^ released by ALG induces calcium signaling and stimulates activation of endothelial cells, NK cells, and fibroblasts in contrast with BIA [19,20], which does not release the same amount of Ca^2+^ as ALG [19]. This difference in the amounts of released Ca^2+^ could explain the differences in the extents of macrophage activation reported in this work. Finally, the presence of toxic contaminants, coming from the marine environment of the algae from which alginate is extracted or introduced during the production process, might explain the reduced cell viability reported with BIA compared with ALG.

## 5. Conclusions

This work demonstrates the interest in calcium-alginate dressings and extracellular calcium for modulation of the macrophage activities necessary to balance the activation and regulation of inflammation. Our results highlight differences between the two calcium-alginate dressings studied in their efficiency to stimulate macrophage pro-inflammatory, as well as pro-repair, functions, both of which are essential to achieve complete wound healing. As previously reported for NK cell activation, the pure calcium alginate (ALG) appears to be the most efficient at restoring the ineffective, perturbed inflammatory balance of chronic wounds. The present results corroborate the efficiency of Algosteril in chronic wound healing, as demonstrated in the clinic, notably in the treatment of diabetic foot ulcers and pressure ulcers [61,62].

## Figures and Tables

**Table 1:** List of primers used for gene expression analysis.

Gene Name	Forward Primer 5′-3′	Reverse Primer 5′-3′
Primers for RT-qPCR		
α-SMA	GGCTGTTTTCCCATCCATTG	TGTCCCATTCCCACCATCA
β2M	TGCTGTCTCCATGTTTGATGTATCT	TCTCTGCTCCCCACCTCTAAGT
GM-CSF	AGGGCCCTTGACCATGA	GGGTTGCACAGGAAGTTTCC
COL1A	GGGCAAGACAGTGATTGAATACAA	ACGTCGAAGCCGAATTCCT
IL-1β	TACCTGTCCTGCGTGTTGAA	TCTTTGGGTAATTTTTGGGATCT
IL-8	AGACAGCAGAGCACACAAGC	ATGGTTCCTTCCGGTGGT
IL-6	TGGCTGAAAAAGATGGATGCT	GATGATTTTCACCAGGCAAGTCT
IL-10	TGCCTTCAGCAGAGTGAAGA	GCAACCCAGGTAACCCTTAAA
TGF-β	GGGAAATTGAGGGCTTTC G	GAACCCGTTGATGTCCACTTG
FABP4	ACGAGAGGATGATAAACTGGTGG	GCGAACTTCAGTCCAGGTCAAC
Primers for Microfluidic qPCR		
(1) Transcription Factors		
STAT1	CAGCTTGACTCAAAATTCCTGGA	TGAAGATTACGCTTGCTTTTCCT
STAT3	ATCACGCCTTCTACAGACTGC	CATCCTGGAGATTCTCTACCACT
STAT6	CGAGTAGGGGAGATCCACCTT	GCAGGAGTTTCTATCAAGCTGTG
SOCS3	AGCAGCGATGGAATTACCTGGAAC	TCCAGCCCAATACCTGACACAGAA
PPARγ	GAC-AGG-AAA-GAC-AAC-AGA-CAA-ATC	GGG-GTG-ATG-TGT-TTG-AAC-TTG
KLF4	CCCACATGAAGCGACTTCCC	CAGGTCCAGGAGATCGTTGAA
MERTK	ACCTCTGTCGAATCAAAGCCC	CTGCACACTGGTTATGCTGAA
JMJD3	CACCCCAGCAAACCATATTATGC	CACACAGCCATGCAGGGATT
AP-1	TCCAAGTGCCGAAAAAGGAAG	CGAGTTCTGAGCTTTCAAGGT
IRF5	GCTGTGCCCTTAACAAGAGC	TGCACCAAAAGAGTAATCCTCAG
MAPK1	TACACCAACCTCTCGTACATCG	CATGTCTGAAGCGCAGTAAGATT
IDO1	GCCAGCTTCGAGAAAGAGTTG	ATCCCAGAACTAGACGTGCAA
TGM2	TGTGGCACCAAGTACCTGCTCA	GCACCTTGATGAGGTTGGACTC
(2) Receptors/Markers M1 or M2		
HLA-DQA1	TCGCTCTGACCACCGTGAT	AGGGACCGTAAAACTGGTACAA
HLA-DQB1	AGACTCTCCCGAGGATTTCGT	GAAGTAGCACATGCCCTTAAACT
HLA-DR A	AGTCCCTGTGCTAGGATTTTTCA	ACATAAACTCGCCTGATTGGTC
CD68	CGAGCATCATTCTTTCACCAGCT	ATGAGAGGCAGCAAGATGGACC
CD86	CTGCTCATCTATACACGGTTACC	GGAAACGTCGTACAGTTCTGTG
CD206	GGGTTGCTATCACTCTCTATGC	TTTCTTGTCTGTTGCCGTAGTT
NOX1	ACCGGGTTTATGATATTCCACCT	GATTTCGACAGACTGGCAAGA
(3) Wound-Healing-Associated Genes		
ARG2	CGCGAGTGCATTCCATCCT	TCCAAAGTCTTTTAGGTGGCAG
LPAR1	CTTTGCTGGGTTGGCCTACTT	GCCATGTGCTAACAGTCAGTCT
LPAR2	ACAGCCCGACTTTCACTTGAG	GCCCACAATGAGCATGACCA
SIPR1	GCCTCTTCCTGCTAATCAGCG	GCAGTACAGAATGACGATGGAG
(4) Cytokines, Chemokines, and MMPs		
CCL15	TCCCAGGCCCAGTTCATAAAT	TGCTTTGTGAGATGTAGGAGGT
CCL20	TGCTGTACCAAGAGTTTGCTC	CGCACACAGACAACTTTTTCTTT
CCL22	ATTACGTCCGTTACCGTCTGC	TCCCTGAAGGTTAGCAACACC
IL-27RA	CCCCGTCTTCGTGAACCTAGA	ACATCTTCGGTAGTGGAACTGG
IL1-ra	CATTGAGCCTCATGCTCTGTT	CGCTGTCTGAGCGGATGAA
IL23A	GAGCCTTCTCTGCTCCCTGATA	GACTGAGGCTTGGAATCTGCTG
IFNG	TAATGCAGGTCATTCAGATGTAG	TGTCACTCTCCTCTTTCCAA
MMP2	CTT-CCA-AGT-CTG-GAG-CGA-TGT	TAC-CGT-CAA-AGG-GGT-ATC-CAT
MMP9	TGTACCGCTATGGTTACACTCG	GGCAGGGACAGTTGCTTCT
(5) Housekeeping Genes		
GAPDH	CCAGGTGGTCTCCTCTGACTTC	GTGGTCGTTGAGGGCAATG
B-actin	TGC-CGA-CAG-GAT-GCA-GAA-G	CTC-AGG-AGG-AGC-AAT-GAT-CTT-GA
β2M	TGCTGTCTCCATGTTTGATGTATCT	TCTCTGCTCCCCACCTCTAAGT

**Table 2:** Upregulated & downregulated DEGs in M1.

M1 Phenotype	Common to All ALG, BIA, & Ca^2+^ 3 mM	Common to Alginates ALG & BIA	Common to ALG & Ca^2+^ 3 mM	Common to BIA & Ca^2+^ 3 mM	ALG Only	BIA Only	Ca^2+^ 3 mM Only
Upregulated genes	**GM-CSF**, **IL-23A, SOCS3**, IL-1ra, **IL-8**	**CCL20**, JMJD3, **AP-1**, LPAR2, **CD86**, **IDO1**	**IL-1β**, **IL-6**		CCL22	**CCL15**, PPARγ	ARG2
Downregulated genes	MerTK, **NOX1**, CD16		HLA-DRA				HLA-DRQB1, **IFN-γ**

**Table 3:** Upregulated & downregulated DEGs in M2a.

M2a Phenotype	Common to All ALG, BIA, & Ca^2+^ 3 mM	Common to Alginates ALG & BIA	Common to ALG & Ca^2+^ 3 mM	Common to BIA & Ca^2+^ 3 mM	ALG Only	BIA Only	Ca^2+^ 3 mM Only
Upregulated genes	**FABP4**, **IL-1ra**, SOCS3, CD86, **ARG2**	**TGM2**, **CCL22**, CCL15		IL-23A		**PPARγ**	IDO1
Downregulated genes	NOX1, **CD163**, HLA-DRQA1, HLA-DRQB1, HLA-DRA	**MerTK**, MMP2		MMP9	**TGF-β**	**CD206**	IFN-γ

**Table 4:** Upregulated & downregulated DEGs in M2c.

M2c Phenotype	Common to All ALG, BIA, & Ca^2+^ 3 mM	Common to Alginates ALG & BIA	Common to ALG & Ca^2+^ 3 mM	Common to BIA & Ca^2+^ 3 mM	ALG Only	BIA Only	Ca^2+^ 3 mM Only
Upregulated genes	CCL20, IL-23A, CD86, **TGM2**, **IL-1ra**		IDO1, **MMP2**		**LPAR1**, CCL15	AP-1, PPARγ	**ARG2**
Downregulated genes	**IL-10**				HLA-DRQB1, HLA-DRA, **SIPR1**		

## Data Availability

The data that support the findings of this study are available from the corresponding author upon reasonable request.

## Supplementary Materials

The following supporting information can be downloaded at: https://www.mdpi.com/article/10.3390/cells14120909/s1, Figure S1: Fibroblast-associated gene expression markers.
