# Supplementary figures and images for: In Vitro Modulation of Macrophage Inflammatory and Pro-Repair Properties Essential for Wound Healing by Calcium and Calcium-Alginate Dressings
Source: Cells. 2025 Jun 16;14(12):909. doi: 10.3390/cells14120909 (PMC12190423; doi:10.3390/cells14120909)

## Supplementary Figure S1

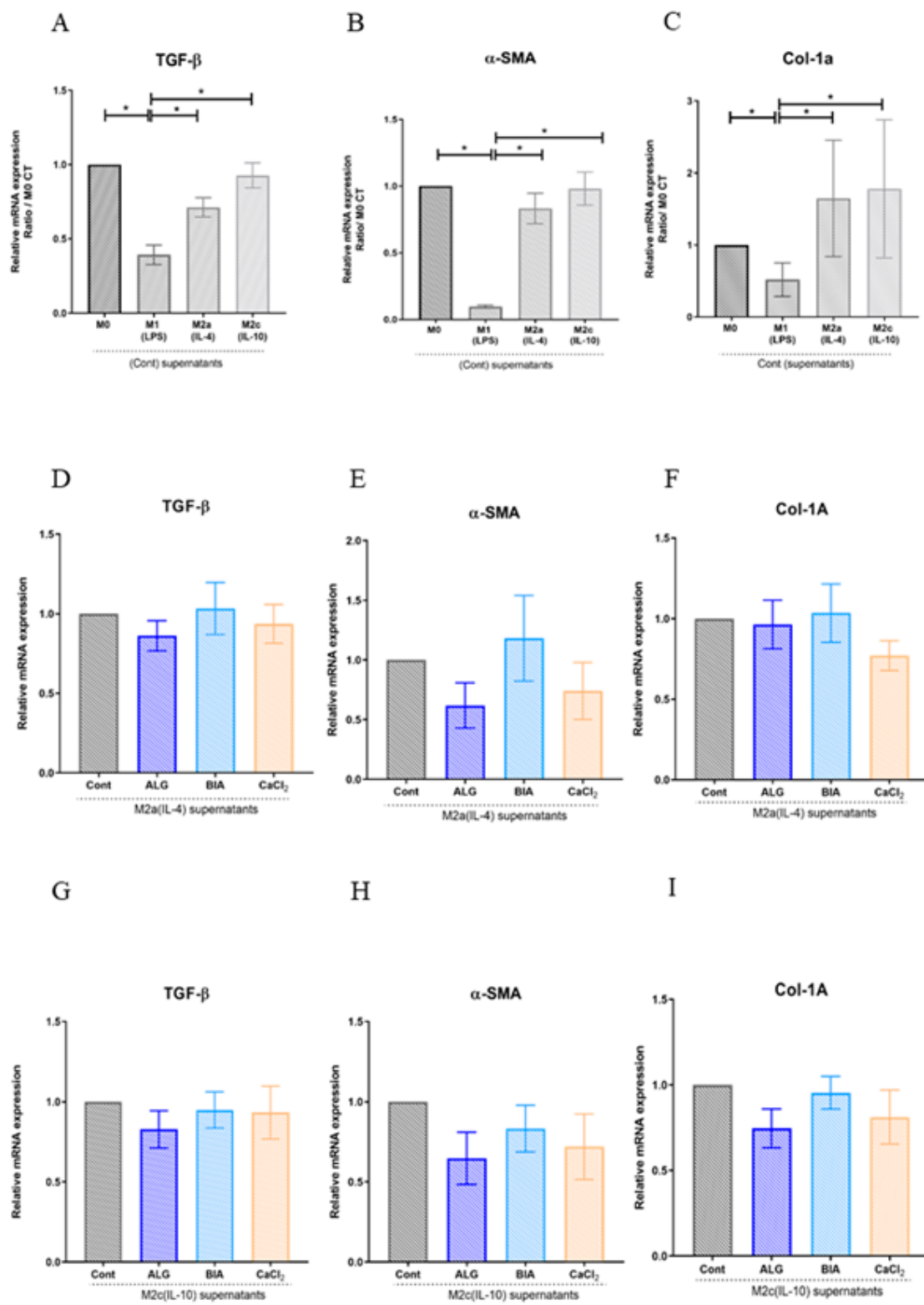

Supplement: Supplementary file 1 [file cells-14-00909-s001.zip › cells-3637337-supplementary.pdf]
